# *Lobosorchis labri* n. sp. (Trematoda: Cryptogonimidae): a host switch from snappers (Lutjanidae) to wrasses (Labridae)

**DOI:** 10.1016/j.ijppaw.2026.101206

**Published:** 2026-02-06

**Authors:** Helen Armstrong, Thomas H. Cribb, Scott C. Cutmore, Salvador Zarco-Perello, Storm B. Martin

**Affiliations:** a Centre for Sustainable Aquatic Ecosystems, Harry Butler Institute, Murdoch University, Murdoch, Western Australia, 6150, Australia; b Biodiversity and Geosciences Program, Queensland Museum, South Brisbane, Queensland, 4101, Australia; c College of Life Sciences and Agriculture, University of New Hampshire, Durham, USA

**Keywords:** Digenea, Labriformes, *Hemigymnus*, Biogeography, Evolutionary history, Diet, Piscivore, Ningaloo reef, Great barrier reef, Coral reef

## Abstract

The first cryptogonimid trematode with adults parasitic in wrasses (Labriformes: Labridae) is proposed. *Lobosorchis labri* n. sp. was recovered at a substantial combined prevalence (63%, 15 of 24) from the thicklipped wrasses *Hemigymnus melapterus* (Bloch) and *H*. *fasciatus* (Bloch) at Ningaloo Reef, Western Australia. Definitive hosts for all other *Lobosorchis* spp. are exclusively tropical snappers, *Lutjanus* spp. (Lutjaniformes: Lutjanidae). Novel material for *Lobosorchis* spp. collected from snappers is also reported, including for *L*. *tibaldiae* Miller & Cribb, 2005 and *L*. *polygongylus* Miller et al., 2009, a new species *L*. *milleri* n. sp., and novel genetic data and host-locality combinations for what are interpreted as uncharacterised species, from Ningaloo Reef, the Great Barrier Reef off eastern Australia, French Polynesia, and Okinawa, Japan. *Lobosorchis labri* has evidently speciated following a host switch from snappers into wrasses. Based on comparison of genetic differences among *Lobosorchis* spp., this switch has occurred recently, in evolutionary terms, yet sufficiently distantly such that *L*. *labri* is now apparently specific for *Hemigymnus* spp. and no longer infects snappers; infections of *Lobosorchis* spp., but not *L*. *labri*, were detected in sympatric snappers at Ningaloo Reef. Cryptogonimids were not detected in our examinations of 98 individuals of *Hemigymnus* spp. on the Great Barrier Reef; *L*. *labri* seemingly does not occur there, and thus the host switch which gave rise to this species likely occurred somewhere in the Indian Ocean. Piscivory in *Hemigymnus* spp. is reported, implied by infection of cryptogonimids and confirmed *via* examination of gut contents.

## Highlights

• First cryptogonimid trematode with sexual adult parasitic in wrasses.
• Host switch of a trematode from snappers to wrasses.
• Two new *Lobosorchis* spp. proposed, and further uncharacterised richness detected.
• Substantial and widespread piscivory reported in *Hemigymnus* wrasses.

## Graphical abstract

**Figure:**
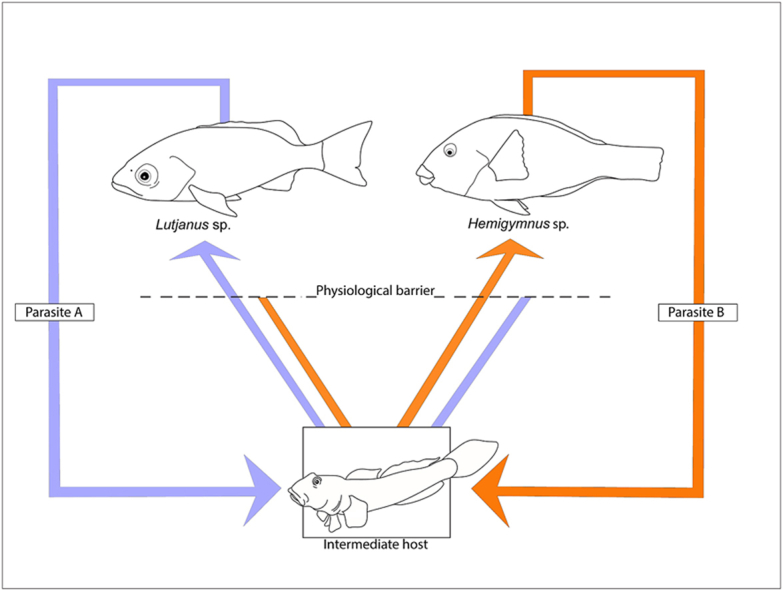

## 1 Introduction

The evolutionary and biogeographical narratives of parasites are intimately intertwined with those of their hosts. Establishment of a parasite lineage in a new host requires sufficient frequency of encounters and subsequent traversal of the physiological compatibility barrier (Euzet and Combes, 1980; Araujo et al., 2015). Not all host switches are equally challenging. Switches between closely related, physiologically similar host species pose minimal compatibility challenges and so should occur frequently provided sufficient encounters take place (de Vienne et al., 2013; D'Bastiani et al., 2023). More dramatic host switches, into substantially phylogenetically and thus physiologically distinct hosts, are less common, but may generate opportunity for subsequent radiation among closely related species of the newly exploited host group (e.g., consider Ziętara and Lumme, 2002 vs Verneau et al., 2023).

The Cryptogonimidae Osborn, 1903 (see Truong et al., 2025 for recent change to recognised family authority) is among the richest families of digenean trematodes, with almost 300 recognised species and over 80 recognised genera (Gibson et al., 2025), in mostly tropical marine and freshwater ecosystems worldwide (Miller and Cribb, 2008). As far as is known, all cryptogonimids use fishes as second intermediate hosts. Definitive hosts include substantially piscivorous fishes, mostly teleosts, but also crocodilians, freshwater and marine snakes, Neotropical freshwater turtles, and giant salamanders (Tkach and Snyder, 2003; Miller and Cribb, 2008; Martínez-Aquino et al., 2017; Cajiao-Mora et al., 2025). The greatest richness of cryptogonimids is concentrated in the Lutjaniformes (Lester and Sewell, 1989; Miller and Cribb, 2007; Miller et al., 2011; Martin and Cutmore, 2022), a grouping of tropical marine teleosts which comprises the snappers and fusiliers (Lutjanidae including Caesioninae), and the grunts and sweetlips (Haemulidae). This order is frequently (but not always) recognised or supported in recent classifications for the bony fishes (e.g., Betancur-R et al., 2017 vs Thacker and Near, 2025).

Here we report on a cryptogonimid consistent with the genus *Lobosorchis* Miller and Cribb, 2005, from thicklipped wrasses (Labriformes: Labridae: *Hemigymnus* spp.). The wrasses are one of the richest families of fishes and are especially important invertivores on coral reefs. Although several lineages of wrasses include species which are substantially piscivorous (see Cowman et al., 2009), none are convincingly known as definitive hosts for cryptogonimids. The genus *Lobosorchis* was proposed for *L*. *tibaldiae* Miller and Cribb (2005) found in two snappers, the Spanish flag snapper *Lutjanus carponotatus* (Richardson) on the Great Barrier Reef, Australia and the blackspot snapper *Lu*. *fulviflamma* (Forsskål) from New Caledonia. Miller et al. (2009) proposed a second species, *L*. *polygongylus* Miller et al., 2010 from the paddletail snapper *Lu*. *gibbus* (Forsskål) from both the Maldives and the Great Barrier Reef, provided genetic sequence data for both *L*. *polygongylus* and *L*. *tibaldiae*, and detected two further putative, undescribed species on the basis of sequence data for metacercariae recovered from smaller reef fishes. The concept of *Lobosorchis* is distinguished from other cryptogonimid genera by the combination of follicular testes, oral spines, and restriction of vitelline follicles to the prebifurcal region of the forebody (Miller and Cribb, 2005).

## 2 Materials and methods

### 2.1 Host and parasite collection

Fishes were collected by spearfishing at Ningaloo Reef in Western Australia, off Lizard Island and Heron Island in the northern and southern Great Barrier Reef, Queensland, respectively, and several locations in French Polynesia, and were purchased from a fish market in Okinawa, Japan. Fishes were dissected fresh. The digestive tract was removed, opened and inspected for helminths under stereo microscope. The gut and its contents were then washed and re-examined as per Cribb and Bray (2010) and Cutmore et al. (2025). Recovered trematodes were fixed in near-boiling saline and preserved in 80% ethanol (Cutmore et al., 2025).

### 2.2 Morphological study

Specimens for morphological study were rehydrated in tap water, stained with Semichon's acetocarmine or Mayer's haemotoxylin, destained in 1% HCl, neutralised in 1% NH_4_OH, dehydrated in a graded ethanol series (50%, 70%, 90%, 95%, 100%, 100%), cleared in methyl salicylate and mounted in Canada balsam. Measurements were taken *via* live feed from a DP71 digital microscope camera fitted with a UCMAD3 adaptor to a BX50 microscope with Nomarski interference contrast (Olympus Inc., Tokyo, Japan) using cellSens Standard v1.13. Measurements are in micrometres (μm) with length followed by width and the mean in parentheses. Egg dimensions and length of oral spines were averaged over ten eggs and spines per specimen. Drawings were traced using a camera lucida and digitised in Adobe Illustrator CC using a drawing tablet (Kamvas 22, Huion, Shenzen, China). Type and voucher material has been archived in the Crustacea and Worms collection of the Western Australian Museum, Perth (WAM), and the Queensland Museum, Brisbane (QM). New taxa have been registered in the Ocean Census Biodiversity Data Platform, and in ZooBank as per article 8.5 of the International Code of Zoological Nomenclature (ICZN, 2012).

### 2.3 Generation of sequence data

Genomic DNA was extracted using a DNeasy blood and tissue kit (QIAGEN, Hilden Germany), mostly from tissue dissected from posterior to the gonads, such that most novel sequence data have corresponding hologenophore voucher specimens (see Pleijel et al., 2008; Cutmore et al., 2025). Sequences were generated for the cytochrome oxidase subunit 1 mitochondrial barcoding gene (*cox*1 mtDNA), and the second transcribed spacer region (ITS2 rDNA) and large subunit gene (28S rDNA) of the ribosome genome. The *cox*1 gene was partially amplified as per Wee et al. (2017) with the primers dig_cox1Fa (5′-ATG ATW TTY TTY TTY YTD ATG CC-3′) and dig_cox1R (5′-TCN GGR TGH CCR AAR AAY CAA AA-3′) and the following cycle schedule: 1 × (3 min, 94 °C), 40 × (30 s, 94 °C; 30 s, 50 °C; 30 s, 72 °C), 1 × (10 min, 72 °C). The complete ITS2 region, together with flanking partial 5.8S and 28S, was amplified with the primers 3S (5ʹ GGT ACC GGT GGA TCA CGT GGC TAG TG 3ʹ, Morgan and Blair, 1995) and ITS2.2 (5ʹ CCT GGT TAG TTT CTT TTC CTC CGC 3ʹ, Cribb et al., 1998) and the following cycle schedule: 1 × (3 min, 95 °C; 2 min, 45 °C; 90 s, 72 °C), 4 × (45 s at 95 °C; 45 s, 50 °C; 90 s, 72 °C), 30 × (20 s at 95 °C; 20 s, 52 °C; 90 s, 72 °C), 1 × (5 min, 72 °C). The 28S gene was partially amplified with the primers LSU5 (5′-TAG GTC GAC CCG CTG AAY TTA AGC-3′, Littlewood, 1994) and 1500R (5′-GCT ATC CTG AGG GAA ACT TCG-3′, Snyder and Tkach, 2001) and the following cycle schedule: 1 × (4 min, 95 °C), 30 × (1 min, 95 °C; 1 min, 56 °C; 2 min, 72 °C), 1 × (1 min, 95 °C; 45 s, 55 °C; 4 min, 72 °C). All PCR reaction volumes comprised 25 μl consisting of 2 μl of unquantified genomic DNA, 2.5 μl 10 × reaction buffer (Fisher Biotec Australia), 6 μl cresol-red dye, 2 μl MgCL_2_ at 25 mM, 1 μl combined deoxyribonucleotide triphosphate (dNTPs) at 4 mM, 1 μl per primer at 10 μM, 0.2 μl *Taq* DNA polymerase at 5.5 units/μl, and 9.3 μl H_2_O. Amplicons were visualised *via* electrophoresis of 1.5% agarose gel stained with SYBR Safe (Invitrogen, California, USA), and purified using the Agencourt AMPure magnetic bead purification system (Beckman Coulter, California, USA). Sanger sequencing was completed in both directions using the amplification primers for each marker, outsourced to the Western Australian State Agricultural Biotechnology Centre (SABC) at Murdoch University, using an ABI Prism™ BigDye v3.1 Cycle Sequencing Kit (Applied Biosystems, California, USA) and an ABI 3730 96 capillary machine. Contiguous sequences were assembled and checked for base polymorphisms in Geneious v10.2.2 (Kearse et al., 2012). Novel sequence data were deposited at GenBank (GB); accession numbers are reported in the taxonomic summaries below.

### 2.4 Molecular analyses

Novel sequence data for *cox*1 mtDNA and ITS2 rDNA were examined for genetic diversity and to investigate species boundaries. These datasets, including previously published ITS2 rDNA sequence data for *Lobosorchis* spp. (no previous data are available for *cox*1 mtDNA), were each aligned using MUSCLE (Edgar, 2004) and subjected to pairwise comparison and unrooted neighbour-joining analyses in MEGA 11 (Tamura et al., 2021), using default parameters and nodal support estimated from 1000 bootstrap replicates. No phylogenetic analyses for 28S rDNA sequence data are included here as several broad cryptogonimid analyses have been published recently, all of which include representative species of *Lobosorchis* (Martínez-Aquino et al., 2017; Kmentová et al., 2020; Martin et al., 2022; Yong et al., 2023; Truong et al., 2025).

### 2.5 Fish diet studies

During parasitological examination at Ningaloo Reef and at Lizard Island, Great Barrier Reef, the gut contents of *Hemigymnus* spp. were checked for remains of prey fishes under stereo microscope. For necropsies at Ningaloo Reef, the gut contents were also preserved in 70% ethanol and prey items were later sorted to a broad taxonomic resolution and quantified.

## 3 Results

### 3.1 Overview

**Fig. 1:**
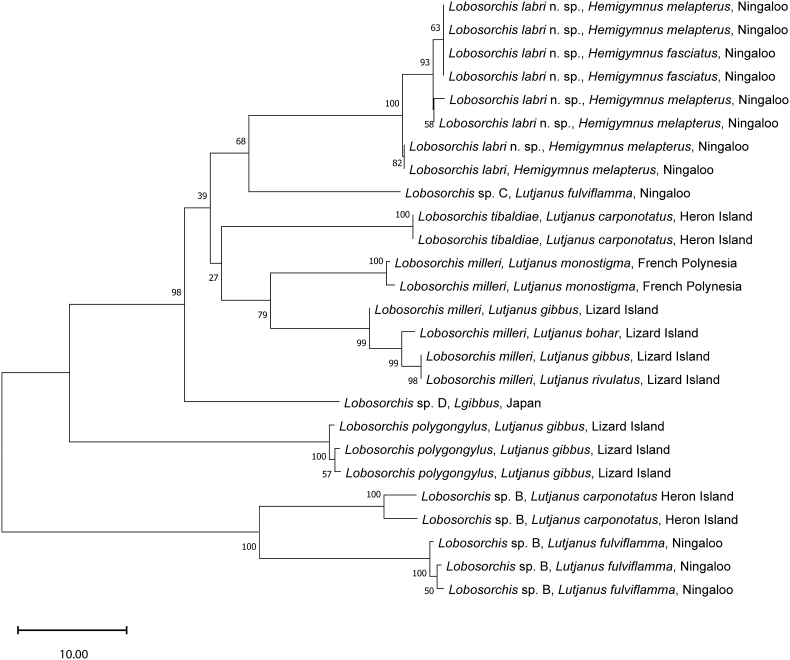
Unrooted neighbour-joining analysis based on partial *cox*1 mtDNA for species of *Lobosorchis*Miller and Cribb (2005) with nodal support estimated from 1000 bootstrap replicates. All sequence data are novel. GenBank accession numbers are provided in the corresponding taxonomic summaries. Scale bar indicates the number of substitutions.

Novel specimens consistent with *Lobosorchis* were collected from the thicklip wrasses *Hemigymnus fasciatus* and *H*. *melapterus* (Labriformes: Labridae) at Ningaloo Reef, as well as from several tropical snappers *Lutjanus* spp. (Lutjaniformes: Lutjanidae) from Ningaloo Reef, the Great Barrier Reef, French Polynesia and Okinawa. Both of the two previously recognised species of *Lobosorchis*, *L*. *tibaldiae* and *L*. *polygongylus*, were recollected, as were sexual adults of *L*. sp. A and *L*. sp. B of Miller et al. (2009), two putatively distinct species previously known only from metacercariae. These four species were identified *via* matching ITS2 rDNA sequence data published previously. Genetic diversity among *Lobosorchis* is limited in the rDNA markers ITS2 and 28S; for example, *L*. *tibaldiae* and *L*. *polygongylus* differ at just two base-positions (bp) in ITS2 rDNA (466 bp total) and at one bp in partial 28S rDNA (855 bp total) sequence data. However, greater levels of genetic diversity were observed among the novel *cox*1 mtDNA (475 bp total) sequence dataset (Table 1, Fig. 1). Thus, the specimens from wrasses are morphologically and genetically distinct from both *L*. *tibaldiae* and *L*. *polygongylus*, and are genetically distinct from *L*. sp. A and *L*. sp. B of Miller et al. (2009); a new species is proposed for these specimens from wrasses below. The novel material and sequence data for *L*. sp. A and *L*. sp. B of Miller et al. (2009) corroborates the previous interpretation that both represent distinct species. The additional material collected allowed for formal characterisation of *L*. sp. A, below, but remains insufficient to characterise *L*. sp. B. In addition to these five species of *Lobosorchis* (i.e., two previously characterised species, two new species, one putative and unnamed species), our genetic prospecting (*cox*1 mtDNA, ITS2 rDNA and 28S rDNA) suggests the presence of two further, uncharacterised species of *Lobosorchis*.

**Table 1:** Pairwise interspecific genetic differences between species of *Lobosorchis*, in number of base-positions (bp), for complete ITS2 rDNA sequence data including flanking 5.8S and 28S (466 bp total) above the diagonal, and partial *cox*1 mtDNA sequence data (475 bp) below the diagonal. Intraspecific genetic variation is included for *cox*1 mtDNA sequence data on the diagonal. For ITS2 rDNA sequence data, intragenomic polymorphic sites are counted as a difference of 0.5 where one of the two nucleotides present is shared (e.g. A and C *vs* A) or 1.0 where neither of the two nucleotides present is shared (e.g., A and C *vs* G).

	*L*. *labri*	*L*. *milleri*	*L*. *polygongylus*	*L*. *tibaldiae*	*L*. sp. B	*L*. sp. C	*L*. sp. D
*L*. *labri*	0–4	3–4.5	4	4	5–6	0	1
*L*. *milleri*	33–40	0–24	1–2	1–2.5	2–4	2–4	1–2.5
*L*. *polygongylus*	57–60	46–57	2	2	4–5	4	3
*L*. *tibaldiae*	37–41	26–36	55–56	0	4–5	4	3
*L*. sp. B	78–84	54–77	68–76	68–75	1–32	5–6	4–5
*L*. sp. C	28–32	37–39	50–51	32	72–73	-	1
*L*. sp. D	33–37	38–44	51–52	35	64–72	38	-

### 3.2 Taxonomy

#### 3.2.1 *Lobosorchis*Miller and Cribb, 2005

*Classification*: Phylum Platyhelminthes Minot, 1876: Class Trematoda Rudolphi, 1808: Subclass Digenea Carus, 1863: Order Plagiorchiida La Rue, 1957: Suborder Opisthorchioidea Looss, 1899: Family Cryptogonimidae Osborn, 1903.

*Synonyms:* none.

*Type-species*: *L*. *tibaldiae*Miller and Cribb (2005).

*Other recognised species: L*. *milleri* n. sp., *L*. *labri* n. sp., *L*. *polygongylus*Miller, Downey & Cribb, 2009.

#### 3.2.2 *Lobosorchis tibaldiae*Miller and Cribb (2005)

*Synonyms*: none.

*Type-host:* Spanish flag snapper *Lutjanus carponotatus* (Richardson) (Lutjaniformes: Lutjanidae).

*Type-locality*: off Heron Island, southern Great Barrier Reef, Queensland Australia.

*Other previous reports: Lu*. *carponotatus* off Lizard Island, northern Great Barrier Reef, and blackspot snapper *Lu. fulviflamma* (Forsskål) from New Caledonia (Miller and Cribb, 2005).

*Novel material:* two immature specimens *ex* 1 of 47 (2%) *Lu*. *carponotatus* collected off Heron Island (23°27′S, 151°55′E) since Miller et al. (2009); not accessioned.

*Novel sequence data:* ITS2 (with flanking 5.8S and 28S): one sequence (submitted to GenBank, PX984852) identical to FJ154899 of Miller et al. (2009); *cox*1 (partial): two identical sequences (GB PX978813).

Remarks: The novel material is identified as *L. tibaldiae via* ITS2 rDNA sequences identical to that provided by Miller et al. (2009), from the same (type) host and locality. *Cox*1 mtDNA sequence data have been generated for the first time.

#### 3.2.3 *Lobosorchis polygongylus* Miller, Downey & Cribb, 2009

*Synonyms*: none.

*Type-host:* humpback red snapper *Lutjanus gibbus* (Forsskål) (Lutjaniformes: Lutjanidae).

*Type-locality*: Rasdhoo Atoll, Maldives.

*Other previous reports: Lu*. *gibbus* off Lizard Island, northern Great Barrier Reef, Queensland, Australia (Miller et al., 2009).

*Novel material:* three specimens, (consumed for sequencing) *ex* 3 of 40 (7.5%) *Lu*. *gibbus* off Lizard Island.

*Novel sequence data:* ITS2 (with flanking 5.8S and 28S): one sequence (GB PX984853) identical to FJ154900 of Miller et al. (2009); *cox*1 (partial): three distinct sequences differing at two bp (0.42%) (GB PX978814–16).

Remarks: Miller et al. (2009) reported identical ITS2 rDNA sequences for specimens identified as *L. polygongylus* from the Maldives and the Great Barrier Reef. Our novel specimens are identified as *L. polygongylus via* identical ITS2 rDNA sequences from a host-locality combination reported by Miller et al. (2009). *Cox*1 mtDNA sequence data have been generated for the first time.

#### 3.2.4 *Lobosorchis labri* n. sp. (Fig. 2A–D)

*Synonyms:* none.

*Type-host:* blackeye thicklip *Hemigymnus melapterus* (Bloch) (Labriformes: Labridae).

*Additional host:* barred thicklip *H*. *fasciatus* (Bloch).

*Type-locality:* vicinity of Five Fingers Reef and Yalobia Passage, approximately 5 km south of Coral Bay township, Ningaloo Reef (23°11′S; 113°46′E), Western Australia.

*Site of infection:* intestine.

*Prevalence:* 10 of 12 (83%) *H*. *melapterus*, 5 of 12 (42%) *H*. *fasciatus*.

*Deposited specimens:* holotype WAM V13589, 13 paratypes WAM V13590–V13600 including two hologenophores WAM V13601–V13602.

*Representative DNA sequences:* ITS2 (with flanking 5.8S and 28S): four identical replicates comprising two from each host species (GB PX984854); 28S (partial): one sequence (GB PX984862); *cox*1 (partial): eight sequences comprising four distinct haplotypes varying at 1–4 bp (0.21–0.84%) and representative of four, two, one and one identical replicates, sequentially (GB PX978817–20).

*ZooBank registration, LSID:* urn:lsid:zoobank.org:act: C93A6FBF-1CEB-441F-B9AE-0E45C8ABDCCB.

*Etymology*: This species is so named because it is the first cryptogonimid known from a labriform definitive host.

*Description* (based on 14 mature specimens). Body small, elongate oval, broadest immediately between ventral sucker and ovary, 707–966 (836) × 415–619 (520), 1.4–1.79 (1.61) times longer than wide. Tegument covered in small, uniform spines. Remnant eyespot pigment usually not apparent. Oral sucker large, wider than long, terminal, opens almost terminally, 40–97 (63) × 106–178 (139); circumoral spines number 48–62 (55), spine length 13–18 (15). Ventral sucker smaller than oral sucker, essentially round, recessed within ventrogenital sac, 80–109 (92) × 87–103 (97); ratio oral/ventral sucker width 1.00–2.25 (1.63). Forebody 190–271 (237), occupies 28% of body length. Prepharynx short, entirely dorsal to oral sucker. Pharynx abuts oral sucker, similar in size to ventral sucker, 86–127 (101) × 91–151 (112); oral sucker/pharynx width ratio 0.91–2.74 (1.7). Oesophagus indiscernible. Intestine bifurcates in forebody immediately posterior to pharynx; intestinal caeca blind, terminate beyond testes near to posterior end of body, 336–646 (553) long or 66% of body length. Testes comprise 6–12 (most frequently 10) discrete follicles, arranged in two orthogonal groups of 3–6 either side of body midline, restricted to hindbody and span from midlevel of ovary almost to caeca termini, overlap caeca ventrally and frequently protrude beyond laterally, often overlap ovary dorsally; individual follicles ellipsoidal, variously subequal but mostly similar, 51–116 (81) × 46–112 (75). Seminal vesicle naked, tubulosaccular with slightly tapered ends, constricted equatorially but not bipartite, arises from vas deferens submedially (usually sinistrally) immediately anterior to ovary, continues to midline at midlevel of ventral sucker, contacts genital pore *via* short, feeble ejaculatory duct. Genital pore median, immediately anterior to ventral sucker, within ventrogenital sac. Gonotyl a prominent, protuberant lobe immediately anterior to genital pore. Ovary deeply lobed, rhombic, medial, essentially restricted to intercaecal zone, immediately anterior to and overlaps testes, separated from ventral sucker by short but distinct gap allowing transverse passage of uterus, 105–193 (152) × 83–212 (147). Seminal receptacle canalicular, subspherical, submedial on opposite side to seminal vesicle (usually dextral), approximately three-quarters size of ovary, anterodorsal to ovary, usually abuts ventral sucker. Laurer's canal not observed. Vitelline follicles distributed in two lateral, usually separate fields constrained to prebifurcal zone, extend posteriorly to level of intestinal bifurcation but never to level of ventral sucker, fill space lateral to pharynx and oral sucker, encroach dorsal to pharynx but usually not to midline; vitelline reservoir small, medial, anteroventral to ovary, drains fields of follicles *via* one vitelline duct each running dorsal to and along either side of ventral sucker. Uterus extensive, fills hindbody but largely occluded by ovary, extends just into forebody either side of ventral sucker to level of intestinal bifurcation, occluded anteriorly by vitelline follicles and does not enter prebifurcal zone. Eggs numerous, small, oval, dark tan, 18–20 (19) × 9–10 (9). Excretory vesicle Y-shaped, dorsal, spans much of intercaecal zone in hindbody, narrows in postcaecal zone, bifurcates dorsal to ovary; arms extend to level of anterior half of pharynx, attenuate in prebifurcal zone. Excretory pore terminal.

*Remarks.* The genus *Lobosorchis* currently comprises two nominal species, *L. tibaldiae* and *L. polygongylus*, both known only from tropical snappers (*Lutjanus* spp.). Those two species are distinguished morphologically by a combination of body size, number of oral spines and number of testes follicles. Using these same characters, *L. labri* is readily distinguishable. Mature specimens are consistently larger than those of *L. tibaldiae* (707–966 μm *vs* 398–565 μm) but comparable to *L. polygongylus* (598–1176 μm), usually have fewer oral spines than *L. polygongylus* (48–62 *vs* 60–81) but overlap substantially with *L. tibaldiae* (47–56), and have testes follicles numbering 6–12, intermediate between *L. tibaldiae* (4–5) and *L. polygongylus* (13–25). *Lobosorchis labri* is also distinguished from both those species by the level at which the fields of vitelline follicles meet the uterus, in the forebody at the level of the intestinal bifurcation *vs* at the midlevel of the ventral sucker in those two species; this has the effect that the uterus enters the forebody only in *L. labri*, and likewise that the vitelline follicles are restricted prebifurcally only in *L. labri.* Genetically, *L. labri* is more distinctive in ITS2 rDNA and 28S rDNA sequences from *L. polygongylus* and *L. tibaldiae* than those species are from each other and is also clearly distinct from those species in *cox*1mtDNA sequences (Table 1).

#### 3.2.5 *Lobosorchis milleri* n. sp. (Fig. 2B and C)

*Synonyms*: *L*. sp. A of Miller et al. (2009).

*Type-host:* humpback red snapper *Lutjanus gibbus* (Forsskål) (Lutjaniformes: Lutjanidae).

*Type-locality*: off Lizard Island, northern Great Barrier Reef, Queensland, Australia.

*Previous reports:* metacercariae in Great Barrier Reef blenny *Ecsenius stictus* (Springer), white-spotted blenny *Salarias alboguttatus* (Kner)*,* and banded jewelled blenny *Salarias fasciatus* (Bloch) (Blenniiformes, Blenniidae)*,* and Bennett’s sharpnose puffer *Canthigaster bennetti* (Bleeker) (Tetraodontiformes, Tetraodontidae), off Lizard Island by Miller et al. (2009).

*Additional host*s *and localities*: Māori snapper *Lu*. *rivulatus* (Cuvier) off Lizard Island, onespot snapper *Lu*. *monostigma* (Cuvier) off Îles Maria (=Nororotu or Hull Island), Austral Islands, French Polynesia (21°48′S; 154°42′W).

*Site of infection:* pyloric caeca.

*Prevalence:* 1 of 40 (2.5%) *Lu*. *gibbus* and 1 of 3 *Lu*. *rivulatus* (33%) off Lizard Island, 1 of 1 *Lu*. *monostigma* off Îles Maria.

*Deposited specimens:* holotype QM G242297, 14 paratypes QM G242298–310, including one hologenophore (QM G242311).

*Novel sequence data*: ITS2 (with flanking 5.8S and 28S): five sequences representative of three unique genotypes (GB PX984855–57), including all three host-locality combinations, with three sequences identical and the other two variable at 1–2 polymorphic bp, longer than but identical (excepting polymorphisms) to FJ154903 of Miller et al. (2009); *28S* (partial): three sequences representative of two unique genotypes with one differing at a single polymorphic base (GB PX984863–64) *ex Lu*. *gibbus* and *Lu*. *rivulatus* at Lizard Island and *Lu*. *monostigma* from Îles Maria; *cox*1: six sequences comprising four representatives of three unique haplotypes differing by 2–3 (0.42–0.63%) bp *ex Lu*. *gibbus* and *Lu*. *rivulatus* at Lizard Island, and two sequences varying at 2 bp *ex Lu*. *monostigma* from Îles Maria (GB PX978821–25) and differing from those at Lizard Island by 22–24 bp.

*ZooBank registration, LSID:* urn:lsid:zoobank.org:act:7A5206A0-5BD0-4686-A6E9-05370E1648A6.

*Etymology*: For Dr Terry Miller, Queensland Museum, to honour his outstanding contribution to taxonomy of the Cryptogonimidae.

*Description* (based on 15 mature specimens). Body small, ovate, broadest slightly at level of ovary, 432–838 (650) × 289–504 (405), 1.49–1.78 (1.59) times longer than wide. Tegument covered in small, uniform spines. Remnant eyespot pigment usually not apparent. Oral sucker wider than long, terminal, opens almost terminally, 47–68 (55) × 102–184 (141); circumoral spines number 45–60 (55), spine length 14–22 (18). Ventral sucker smaller than oral sucker, essentially round, recessed within ventrogenital sac, 54–75 (63) × 61–89 (72). Ratio oral/ventral sucker width 1.09–1.63 (1.31). Forebody 194–263 (224), occupies 32% of body length. Prepharynx short, entirely dorsal to oral sucker. Pharynx abuts oral sucker, slightly larger than ventral sucker, 64–107 (86) × 66–103 (81); oral sucker/pharynx width ratio 1.19–1.81 (1.48). Oesophagus extremely short. Intestinal bifurcation in forebody immediately posterior to pharynx; intestinal caeca blind, terminate beyond testes near to posterior end of body, 298–509 (424) long or 65% of body length. Testes multiple, discrete follicles totalling 13–20 (16) in two orthogonal groups of 5–10 (8) either side of body midline, restricted to hindbody, overlap caeca ventrally and extend laterally near to body margins, often overlap ovary dorsally; individual follicles ellipsoidal, variously subequal but mostly similar, 50–75 (65) × 51–89 (73). Seminal vesicle naked, tubulosaccular with slightly tapered ends, constricted equatorially but not bipartite, arises from vas deferens submedially (usually dextral) immediately anterior to ovary, continues to midline at midlevel of ventral sucker, communicates with genital pore *via* short, feeble ejaculatory duct. Genital pore median, immediately anterior to ventral sucker, within ventrogenital sac. Gonotyl discrete, protuberant lobe immediately anterior to genital pore. Ovary deeply lobed, medial, essentially restricted to intercaecal zone, immediately anterior to and overlaps testes, separated from the ventral sucker by short but distinct gap allowing transverse passage of uterus, 108–165 (133) × 120–193 (145). Seminal receptacle canalicular, subspherical, submedial on opposite side to seminal vesicle (usually sinistral), approximately half the size of ovary, anterodorsal to ovary, usually abuts ventral sucker. Laurer's canal not observed. Vitelline follicles distributed in two lateral, confluent fields constrained to forebody, extend posteriorly to reach level of anterior margin of gonotyl, extend anteriorly to midlevel of pharynx but usually separated from oral sucker, span width of forebody and fill space between pharynx and ventral sucker; vitelline reservoir small, medial, anteroventral to ovary, drains fields of follicles *via* one vitelline duct each running dorsal to and along either side of ventral sucker. Uterus extensive, fills hindbody but largely occluded by ovary, does not extend into forebody, occluded anteriorly by vitelline follicles and does not enter prebifurcal zone. Eggs numerous, small, oval, dark tan, 17–19 (18) × 7–9 (8). Excretory vesicle Y-shaped, dorsal, spans much of intercaecal zone in hindbody, narrows in postcaecal zone, bifurcates dorsal to ovary; arms extend to level of anterior half of pharynx, attenuate in prebifurcal zone. Excretory pore terminal.

*Remarks*: Using the same characters which distinguish *L*. *labri* from *L*. *polygongylus* and *L*. *tibaldiae* above, *L*. *milleri* is also readily distinguishable. Mature specimens are mostly smaller than those of *L*. *labri* (432–838 μm *vs* 707–966 μm), but overlap substantially with both *L*. *tibaldiae* (398–565 μm) and *L. polygongylus* (598–1176 μm), have fewer oral spines than *L*. *polygongylus* (45–60 *vs* 60–81), but overlap substantially with *L*. *tibaldiae* (47–56) and *L*. *labri* (48–62), and have testes follicles numbering 13–20, more than in *L*. *tibaldiae* (4–5) and *L*. *labri* (6–12), but completely within the range of *L*. *polygongylus* (13–25). The distribution of the fields of vitelline follicles is similar in *L*. *milleri* to both *L*. *tibaldiae* and *L*. *polygongylus*. Miller et al. (2009) detected this species only as metacercariae, yet concluded it likely represents a distinct species based on ITS2 rDNA sequence data. They generated five identical replicate sequences, which differ from those for each of *L*. *tibaldiae* and *L*. *polygongylus* at just one (different) bp each. Our novel ITS2 rDNA and *cox*1 mtDNA sequence data corroborate their interpretation (Table 1). We generated five replicate ITS2 rDNA sequences essentially identical to their sequences, three from Lizard Island and two from French Polynesia and differing only at 1–2 polymorphic bp. In *cox*1 mtDNA sequences, these same specimens differ by 22–24 (4.63–5.05%) bp over this geographic range, fewer than interspecific differences between *L*. *milleri* and *L*. *polygongylus* in sympatry at Lizard Island (54 bp or 11.37%), and between those species and *L*. *tibaldiae* from Heron Island on the southern Great Barrier Reef (36–37 bp or 7.58–7.79%).

#### 3.2.6 *Lobosorchis* sp. B of Miller et al. (2009)

*Previous reports:* metacercariae in white damselfish *Dischistodus perspicillatus* (Cuvier) (Pomacentridae) off Lizard Island by Miller et al. (2009).

*Novel material:* two specimens (consumed for sequencing) *ex* 2 of 18 *Lu*. *carponotatus* off Heron Island, and one specimen (consumed for sequencing) *ex* 1 of 3 (33%) *Lu*. *fulviflamma* at Ningaloo Reef.

*Novel sequence data*: ITS2 (with flanking 5.8S and 28S): one sequence from Heron Island (GB PX984858) and one sequence from Ningaloo Reef (GB PX984859) substantially longer than and differing at one bp and one polymorphic bp, respectively, from FJ154904 of Miller et al. (2009); 28S (partial): one sequence from Heron Island (GB PX984865) *cox*1: five distinct sequences (GB PX978826–30).

*Remarks*: Like *L*. *milleri* above, *L*. sp. B was considered a putatively distinct species by Miller et al. (2009) based on ITS2 rDNA sequence data, generated from a single metacercaria. Indeed, it is the most genetically distinct species among all the *Lobosorchis* material, both in ITS2 rDNA and *cox*1 mtDNA sequence data. As for *L*. *milleri* above, our new material for *L*. sp. B are sexual adults, but are insufficient to characterise this species. Here, *L*. sp. B were detected from the type-host and type-locality for *L*. *tibaldiae*. This raises the possibility that the type-material for *L*. *tibaldiae* included specimens of *L*. sp. B, although we think this unlikely because *L*. *tibaldiae* is more similar to *L*. *polygongylus* genetically yet those two species are easily distinguished morphologically. The novel material identified as *L*. sp. B from Ningaloo Reef differs by 30–32 bp (6.32–6.74%) in *cox*1 mtDNA sequence data relative to the material from the Great Barrier Reef, similar to intraspecific variation for *L*. *milleri* between the Great Barrier Reef and French Polynesia. Specimens of *L*. sp. B from Ningaloo Reef and the Great Barrier Reef differ in ITS2 rDNA sequence data by 0–1 bp.

#### 3.2.7 *Lobosorchis* sp. C

*Novel material:* one specimen (consumed for sequencing) *ex* 1 of 3 *Lu*. *fulviflamma* at Ningaloo Reef.

*Novel sequence data*: ITS2: one sequence (GB PX984860). *Cox*1: one sequence (GB PX978831).

*Remarks*: We hypothesise that the above material represents another distinct species of *Lobosorchis* based on genetic data but for which insufficient material prevents morphological comparison or formal characterisation. *Lobosorchis* sp. C is identical to *L*. *labri* in ITS2 rDNA sequence data but differs at 32 bp (6.74%) in *cox*1 mtDNA sequence data. We interpret this distinction as being consistent with interspecific variation. Although this distinction is comparable to what is interpreted here as intraspecific variation within *L*. *milleri* between Lizard Island and French Polynesia, and within *L*. sp. B between Heron Island and Ningaloo Reef, it is also comparable to the interspecific difference between *L*. *milleri* and *L*. *tibaldiae*, and the distinction here is in sympatry and substantially greater than within the eight replicate sequences for *L*. *labri* (0–4 bp or 0–0.84%).

#### 3.2.8 *Lobosorchis* sp. D

*Novel material:* one specimen (consumed for sequencing) *ex* 1 of 1 *Lu*. *gibbus* from Mizugawa fish market, Okinawa, Japan.

*Novel sequence data*: ITS2: one sequence (GB PX984861) *cox*1: one sequence (GB PX978832)

*Remarks*: The specimen recovered from Japan again suggests an apparently distinct species requiring further collection. *Lobosorchis* sp. D is distinct in both ITS2 rDNA and *cox*1 mtDNA sequence data (see Table 1). Considering identical ITS2 rDNA sequence data has been demonstrated over considerable geographic range for *L*. *polygongylus* and *L*. *milleri*, it seems unlikely that the specimen from Japan will prove to represent any of the *Lobosorchis* spp. known from elsewhere. No morphological study was possible.

### 3.3 Diet analysis of *Hemigymnus* spp

Gut contents from 12 *H*. *fasciatus* and 12 *H. melapterus* from Ningaloo Reef were examined. Of these, 10 (42%) had remains of prey fishes. At Lizard Island, 21 guts were examined (one *H. fasciatus*, 20 *H. melapterus*), from which 2 (10%) had remains of prey fishes (Fig. 3). Almost all prey fish remains were well digested with only scales and bones remaining; one had remains that included flesh. The gut content from *Hemigymnus* spp. collected from Ningaloo Reef in austral winter 2022 had a higher percentage of prey fish, in 9 of 12 (75%), than in the austral summer 2023 from which only 1 of 12 (8.3%) contained prey fish. Fish remains comprised approximately 5% of prey items discernible in gut content for both *H*. *fasciatus* and *H*. *melapterus*. Brachyurans and gastropods comprised the largest component of stomach contents in both *Hemigymnus* spp.

**Fig. 2:**
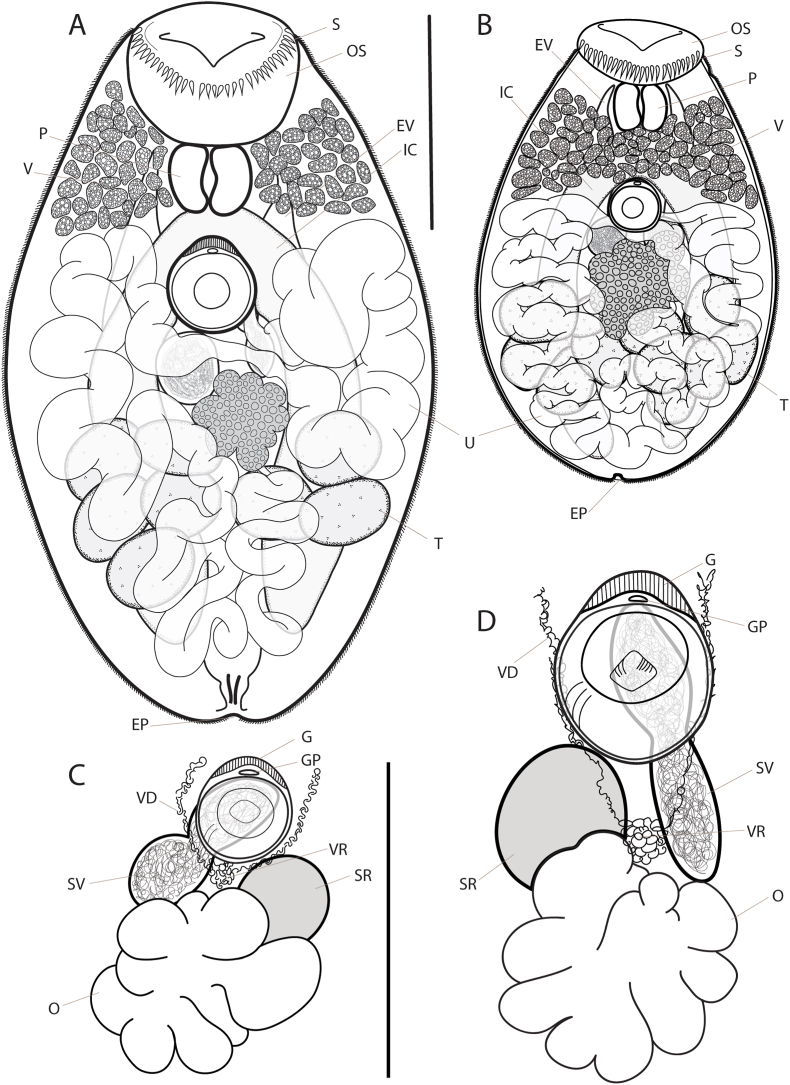
Ventral perspectives of holotypes, to scale, for A. *Lobosorchis labri* n. sp., from the intestine of *Hemigymnus melapterus* at Ningaloo Reef, Western Australia (WAM V13589), and B. *Lobosorchis milleri* n. sp. from a pyloric caecum of *Lutjanus gibbus* at Lizard Island, northern Great Barrier Reef, Queensland (QMXXXX). Terminal male genitalia and ovarian complex, with uterus omitted, to scale, for C. *L*. *milleri* and D. *L*. *labri*. EV, excretory vesicle; EP, excretory pore; G, gonotyl; GP, genital pore; IC, intestinal caeca; O, ovary; OS, oral sucker; P, pharynx; S, oral sucker spines; SP, seminal receptacle; SV, seminal vesicle; T, testes; U, uterus; V, vitellarium; VD, vitelline duct; VR, vitelline reservoir. Scale-bars: A–B 240 μm, C–D 50 μm.

**Fig. 3:**
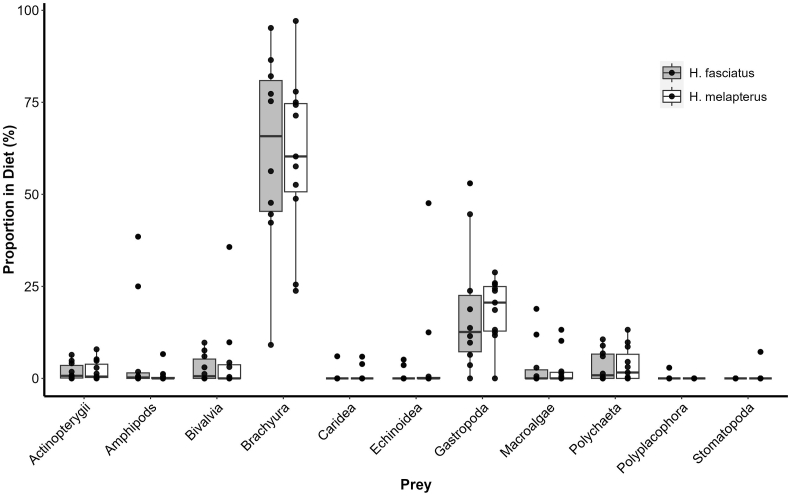
Diet items identified in gut contents of *Hemigymnus* spp. Dark grey boxes represent diet items of *H*. *fasciatus* and white boxes represent *H*. *melapterus*. The largest proportion of both species' diets are true crabs (Brachyura) followed by sea snails (Gastropoda). All other diet items contribute <10% of the fish diet, including prey fish species. There is significant overlap of all recorded *Hemigymnus* spp. diet contents with both displaying similar percentages of the same prey items.

## 4 Discussion

### 4.1 Richness and biogeography of *Lobosorchis*

This investigation has increased the known richness of *Lobosorchis* spp., from four species, two characterised and two putative, to seven species, four characterised and three putative. This increased richness is a consequence of expanded sampling in the Indo-West Pacific beyond the Great Barrier Reef, which has been subjected to far more intensive genetic prospecting for marine fish-trematodes than anywhere else in the region (Cribb et al., 2016; Cutmore et al., 2025). The three additional species detected here were from Ningaloo Reef in Western Australia, and Okinawa, Japan. Nevertheless, one known species from the Great Barrier Reef, *Lobosorchis* sp. B of Miller et al. (2009), remains uncharacterised. This is remarkable, because, among that effort on the Great Barrier Reef, several *Lutjanus* spp., including *Lu*. *carponotatus* and *Lu*. *fulviflamma*, the only two known definitive hosts for *L*. sp. B, are among the most intensively examined of fishes. The elusiveness of *Lobosorchis* sp. B, in particular, reflects the difficulties of investigating *Lobosorchis*, which comprises among the smallest of the diverse cryptogonimids in tropical snappers, and which seemingly occur only in some of the available snapper species, and at modest prevalence and intensity.

Each of *Lobosorchis polygongylus*, *L*. *milleri* and *L*. sp. B has a broad geographical range in the Indo-West Pacific, spanning from the Great Barrier Reef, where all three occur, to the Maldives, French Polynesia and Ningaloo Reef, respectively. Corroboration of morphology with inference from the ribosomal genetic markers has been critical in determining conspecificity across these geographic ranges. Conversely, the low levels of interspecific variation in the ribosomal markers, though useful over geographic range, risks underestimating total species richness in this system if used in isolation; *L*. *labri* and the putative *L*. sp. C are identical in ITS2 rDNA sequence data but clearly separated by *cox*1 mtDNA sequence data in sympatry, and the presence of intragenomic polymorphisms in ITS2 rDNA sequence data for *L*. *milleri* causes difficulty delineating this species based on this marker alone, though again it is clearly separated in *cox*1 mtDNA sequence data. Neither *L*. *polygongylus* nor *L*. *tibaldiae* has been detected at Ningaloo Reef, but sampling of *Lutjanus* spp. there remains insufficient to conclude these species do not occur there. Conversely, *L*. *labri* and *L*. sp. C have so far been detected only at Ningaloo Reef and sampling of the relevant hosts on the Great Barrier Reef is sufficient to conclude that both seemingly do not occur there: we (Cribb and colleagues over recent decades including intensive investigations for cryptogonimids by Miller and colleagues, see also summary of effort by Cutmore et al., 2025) have examined 78 *Lutjanus fulviflamma* and 98 *Hemigymnus* spp. there, specifically at Heron Island and Lizard Island. These fishes all have broad ranges in the tropical Indo-West Pacific; evidently, the range for both *L*. *labri* and *L*. sp. C, though incompletely known, are lesser than for those of their definitive hosts.

### 4.2 First cryptogonimid from wrasses

*Lobosorchis labri* is remarkable because it exploits labrids as definitive hosts, and seemingly does not exploit lutjanids. Indeed, *Lobosorchis labri* is the only cryptogonimid convincingly known to infect labriform fishes as definitive hosts. The only previous report of cryptogonimid sexual adults from a wrasse is that of Sekhar and Threlfall (1970), who found two specimens identified as a potentially undescribed species consistent with the concept of *Metadena* in 1 of 808 (∼0.1%) *Tautogolabrus adspersus* (Walbaum), a mostly invertivorous wrasse restricted to the Northwest Atlantic (Bender et al., 2025). Although there is no reason to doubt their identification, the reported prevalence might suggest that this infection was incidental; this cryptogonimid has not been reported since.

Although *Lobosorchis labri* is the only characterised cryptogonimid from a labrid definitive host, it is not the first digenean to be reported from both labrids and lutjanids. Indeed, several species are reportedly shared between fishes of these families, including: *Deretrema fusillus* (Linton, 1910) (Zoogonidae), *Hamacreadium mutabile* (Linton, 1910), *Helicometra fasciata* (Rudolphi, 1819), *Helicometrina execta* (Linton, 1910) and *Helicometrina nimia* (Linton, 1910) (Opecoelidae), *Lecithochirium floridense* (Manter, 1934), *Lecithochirium microstomum* (Chandler, 1935), *Lecithochirium monticellii* (Linton, 1898) and *Lecithochirium musculus* (Looss, 1907) (Hemiuridae), and *Schikhobalotrema acutum* (Linton, 1910) (Haplosplanchnidae). For none of these species are genetic sequence data available from both the lutjanid and labrid definitive hosts, and most are species represented by numerous records spanning dubiously broad host and geographic ranges. Nevertheless, all these species, except *Hamacreadium mutabile* and *Schikhobalotrema acutum* (see Martin et al., 2017; Pérez-Ponce de León et al., 2024, respectively), are plausibly shared between snappers and wrasses, or at least plausibly representative of congeneric taxa collectively exploiting snappers and wrasses, because most of these species likely utilise crustacean intermediate hosts, which are common prey (unlike fishes) for both snappers and wrasses.

Indeed, *Lobosorchis labri* is among the few digeneans known from wrasses which are presumably transmitted through piscivory; other examples include the unusual and monotypic acanthocolpid *Venusicola inusitatus* Bray and Cribb (2000) from the Venus tuskfish *Choerodon venustus* (De Vis), and, most remarkably, *Rhipidocotyle labroidei* Jones et al., 2003 (Bucephalidae), a parasite of the cleaner wrasse *Labroides dimidiatus* (Valenciennes) presumably transmitted when the cleaner cheats client fishes by taking bites of tissue (Bray and Cribb, 2000; Jones et al., 2003). Furthermore, those wrasses particularly prone to piscivory (e.g., *Cheilinus*, *Epibulus*, *Oxycheilinus* spp.) support a depauperate digenean fauna, to the extent to which they have been examined (see e.g., Muñoz et al., 2006).

### 4.3 Piscivory in thicklipped wrasses

Most previous diet inferences for *Hemigymnus* spp. have been based on observation of feeding behaviour, which is unique and distinctive in these fishes: prey are sorted from mouthfuls of sand and gravel (Randall, 2013). To our knowledge, gut contents have been reported previously for these fishes in two studies, though both were based on mostly small individuals (Hiatt and Strasburg, 1960; Sano et al., 1984). Even so, Sano et al. (1984) reported that fish comprised 2% of diet items in their three largest specimens of *H*. *melapterus* (108–178 mm SL) from Okinawa, Japan. The prevalence of fish material observed here among the gut content of both species of *Hemigymnus* at Ningaloo Reef and the Great Barrier Reef clearly demonstrates that these species regularly prey on small fishes, and that this feeding behaviour is geographically widespread, at least across their range in Australian waters. The prevalence of fish material in the gut contents suggests ingestion of fishes is unlikely to be accidental. The phylogeny of wrasses suggests that piscivory in *Hemigymnus* spp. is novel and arose independent of piscivory in other wrasses (Cowman et al., 2009; Hughes et al., 2023). We suspect the prey fishes are cryptobenthic species (see Brandl et al., 2018) rather than small demersal fishes, but our attempts to identify prey fish through simple genetic amplification failed, and we gleaned no further information to indicate whether various *versus* specific fishes are typical prey, nor whether prey fishes are likely to be benthic *versus* fossorial; inference from the life cycle of *Lobosorchis* spp. is limited because it is generally presumed that specificity for the second-intermediate hosts is low (Cribb et al., 2003; Miller et al., 2011).

Fish material comprised only about 5% of the diet items in the gut content of *Hemigymnus* spp. at Ningaloo Reef. Fish prey are likely relatively large and nutritious compared to other diet items, and so our method perhaps fails to capture the importance of fish in the diet. Regardless, it is clear that fishes are taken frequently but in low numbers relative to other prey. Importantly, it is also clear that, even as a minor diet item, these prey fishes are sufficient to maintain the life cycle of *L*. *labri* at high prevalence. Comparable examples of trophic transmission *via* seemingly minor diet items in coral reef fishes includes species of *Polypipapiliotrema* Martin, Cutmore & Cribb, 2018 (Opecoelidae: Polypipapiliotrematinae), which are transmitted from coral polyps to butterflyfish species (Chaetodontidae) considered obligate corallivores as well as those considered infrequent coral predators (Martin et al., 2018). Similarly, Wee et al. (2020) speculated that *Retroporomonorchis pansho* Wee et al. (2020) (Monorchiidae), one of only two monorchiids convincingly known from lutjanids, is likely maintained in the blacktail snapper *Lutjanus fulvus* (Bloch & Schneider) *via* minor yet sufficient predation on bivalves, the dominant second-intermediate host group for monorchiids and atypical prey for most tropical snappers. These cases, as here, involve degrees of uncertainty due to scant understanding of both the specific fish diets and the specific parasite life cycles.

### 4.4 The host switch timeline

*Lobosorchis labri* is evidently an example of a significant host switch, from a lutjanid to a labrid. The high prevalence of *L*. *labri* detected in *Hemigymnus* spp. precludes any interpretation of these infections as incidental. The direction of the host switch is unambiguous considering that: (1) *L*. *labri* appears to be derived *vs* basal among *Lobosorchis* spp. as the intergenetic distance for *cox*1 mtDNA sequence data between *L*. *labri* and *L*. sp. C, in sympatry at Ningaloo Reef, is among the smallest of the available comparisons, (2) known definitive hosts for all other *Lobosorchis* spp., including the uncharacterised diversity uncovered by molecular prospecting here, are exclusively *Lutjanus* spp., and (3) the broader context of substantial diversity and richness of cryptogonimids in lutjaniform fishes (see recent phylogenetic reconstructions in Martínez-Aquino et al., 2017; Kmentová et al., 2020; Martin and Cutmore, 2022; Yong et al., 2023; Truong et al., 2025).

**Fig. 4:**
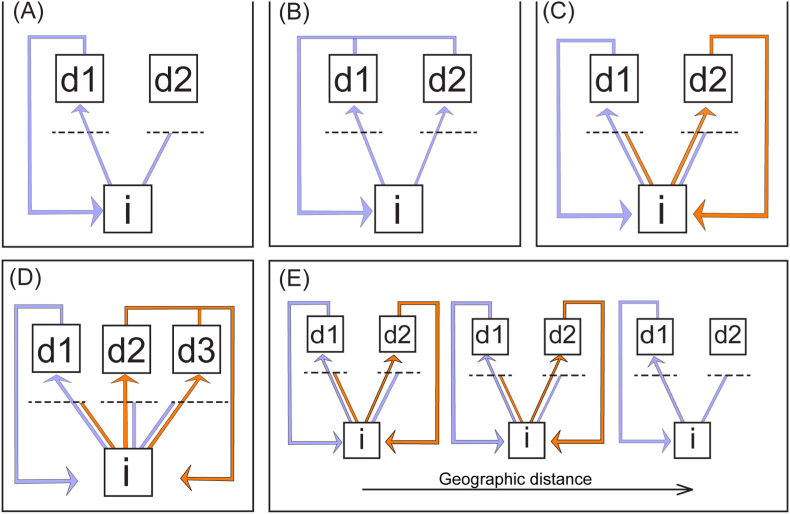
Theoretical representation of the sequential steps (A–C) and possible consequences (D and E) in a definitive host switch for a life cycle with trophic transmission. Hosts are represented by boxes and populations of parasites by coloured lines; the box (i) represents any number of intermediate hosts in the life cycle. The dashed line indicates the physiological barrier which must be crossed to establish infection. A. The state prior to the host switch, where the parasite encounters but does not infect host (d2). B. Adoption of host (d2) *via* breach of the physiological barrier with sufficient frequency. C. Speciation of the parasite and separation from the original definitive host (d1). Note that the intermediate hosts now support both parasites and that both parasites continue to encounter both definitive hosts. D. Radiation: host (d3) is physiologically similar to (d2) and thus a subsequent host switch may occur. E. Geographic extension: supposing that the hosts and the original parasite all occur over a geographic range, both adoption of the novel host and speciation of the parasite occur somewhere within that range and may then spread, or not.

We hypothesise that the novel host adoption and subsequent speciation event that gave rise to *L*. *labri* each occurred in the Indian Ocean because the species seemingly does not occur in *Hemigymnus* spp. on the Great Barrier Reef. Given that *L*. *labri* remains morphologically and genetically conserved among *Lobosorchis* spp., the adoption of the wrasse hosts presumably occurred recently, in an evolutionary context. Nevertheless, the event was sufficiently long ago such that subsequent speciation has occurred, and such that *L*. *labri* perhaps no longer exploits lutjanids (based on modest sampling of lutjanids at Ningaloo Reef). All three events, namely the novel adoption, speciation, and separation from the original host, must occur at some theoretical place and time, but these events are not instantaneous, because genetic changes spread through populations (see Fig. 4). For example, the apparent absence of *L*. *labri* on the Great Barrier Reef might suggest a lag between the host switch occurring somewhere in the Indian Ocean and propagation of the new species across the range of the definitive hosts. Although first intermediate molluscan hosts are unknown for *Lobosorchis* spp., we presume all the appropriate hosts for *L*. *labri* are present on the Great Barrier Reef, given than other species of *Lobosorchis* occur there and that there is no reason to suspect the detected switch in definitive hosts would be followed (or preceded) by a switch in molluscan hosts. Importantly, following speciation, metacercariae of *L*. *labri* presumably continue to encounter snappers with high frequency, and, likewise, metacercariae of the various *Lobosorchis* spp. in snappers, as well as other cryptogonimids and indeed other digeneans with fish intermediate hosts, presumably encounter *Hemigymnus* spp. with comparably high frequency. These considerations attest to the rarity and significance of host switches as dramatic as that detected here.

The legacy of a host switch is the potential for subsequent radiation. Because physiological barriers between host species within a group are lesser than those between groups, the initial switch into a novel host group has the potential to represent a key innovation facilitating further radiation within that group (e.g., see Ziętara and Lumme, 2002), provided no encounter barrier prevents this possibility. In the case of *L*. *labri*, the switch from a lutjanid to a labrid traverses considerable phylogenetic and thus presumably physiology distinction, and the Labridae is a family of great richness and thus, it would seem, great opportunity. However, substantially piscivorous wrasses, that is, those wrasses which likely encounter *L*. *labri* metacercariae, are few, and scattered across the labrid phylogeny such that none are especially closely related to *Hemigymnus* spp. (Cowman et al., 2009). The closest substantially piscivorous relatives are the ring wrasses, *Hologymnosus* spp., from none of which are any digeneans, or indeed any parasites known—they have likely never been intensively examined. Given that no piscivorous wrasses are closely related to *Hemigymnus* spp. and that the host switch which gave rise to *L*. *labri* is seemingly recent, we predict that no subsequent radiation has occurred in wrasses beyond the small *Hemigymnus* genus. Although wrasses are a diverse group which remain far from well-sampled, we think there is reasonable possibility that *L*. *labri* might indeed prove to be the only cryptogonimid with labriform definitive hosts worldwide.

## CRediT authorship contribution statement

**Helen Armstrong:** Writing – review & editing, Writing – original draft, Visualization, Project administration, Investigation, Funding acquisition, Formal analysis, Conceptualization. **Thomas H. Cribb:** Writing – review & editing, Supervision, Investigation, Funding acquisition, Conceptualization. **Scott C. Cutmore:** Writing – review & editing, Supervision, Resources, Project administration, Investigation, Funding acquisition, Formal analysis. **Salvador Zarco-Perello:** Writing – original draft, Visualization, Investigation, Funding acquisition, Formal analysis. **Storm B. Martin:** Writing – review & editing, Writing – original draft, Visualization, Supervision, Resources, Investigation, Funding acquisition, Formal analysis, Conceptualization.

## Funding

This work was supported by the 10.13039/501100001138Australian Biological Resources Study (National Taxonomy Research Grants ABRS NTRG G046WN7 and 4-H04JDSM); the Australia and Pacific Science Foundation (APSF21048), the Nippon Foundation: Nekton Ocean Census programme (https://oceancensus.org ); the Royal Society of Western Australia (John Glover Research Support Grant); the Society of Australian Systematic Biologists (student travel grant); and the Centre for Sustainable Aquatic Ecosystems, Harry Butler Institute, Murdoch University.

## Declaration of competing interest

The authors declare the following financial interests/personal relationships which may be considered as potential competing interests:S.B. Martin, S.C. Cutmore, T.H. Cribb reports financial support was provided by Australian Government Australian Biological Resources Study. S.B. Martin reports financial support was provided by Australia and Pacific Science Foundation. S.B. Martin reports financial support was provided by Nekton Foundation. H. Armstrong reports financial support was provided by Royal Society of Western Australia. H. Armstrong reports financial support was provided by Society of Australian Systematic Biologists. Serving in an editorial capacity for IJP-PAW, S.B. Martin and T.H. Cribb. Given their role as associate editors, S.B. Martin and T.H. Cribb had no involvement in the peer review of this article and had no access to information regarding its peer review. Full responsibility for the editorial process for this article was delegated to another journal editor. If there are other authors, they declare that they have no known competing financial interests or personal relationships that could have appeared to influence the work reported in this paper.
